# Inoculation with extreme endophytes improves performance and nutritional quality in crop species grown under exoplanetary conditions

**DOI:** 10.3389/fpls.2023.1139704

**Published:** 2023-06-22

**Authors:** Marco A. Molina-Montenegro, Victor M. Escobedo, Cristian Atala

**Affiliations:** ^1^ Centre for Integrative Ecology, Instituto de Ciencias Biológicas, Universidad de Talca, Talca, Chile; ^2^ Centro de Investigación en Estudios Avanzados del Maule (CIEAM), Universidad Católica del Maule, Talca, Chile; ^3^ Instituto de Investigación Interdisciplinaria (I^3^), Universidad de Talca, Talca, Chile; ^4^ Instituto de Biología, Facultad de Ciencias, Pontificia Universidad Católica de Valparaíso, Valparaíso, Chile

**Keywords:** extremophiles, astrobiology, Atacama Desert, endophytes, crops, space farming

## Abstract

**Introduction:**

Technological advances have made possible long space travels and even exoplanetary colonies in the future. Nevertheless, the success of these activities depends on our ability to produce edible plants in stressful conditions such as high radiation, extreme temperatures and low oxygen levels. Since beneficial microorganisms, such as fungal endophytes from extreme environments, have helped agriculture cope with those difficulties, endophytic fungi may be a putative tool to ensure plant growth under exoplanetary conditions. Additionally, growing crops in polyculture has been shown to increase productivity and spatial efficiency, which is essential given the likely space restrictions in such conditions.

**Methods:**

We evaluated the effect of the inoculation with a mix of two fungal endophytes from the Atacama Desert on performance (survival and biomass) and nutritional quality of three crop species (lettuce, chard and spinach) grown under exoplanetary conditions. In addition, we measured the amount of antioxidants (flavonoids and phenolics) as possible mechanisms to cope with such abiotic conditions. The exoplanetary conditions were; high UV radiation, low temperature, low water availability, and low oxygen levels. These crops were put in growing chambers in monoculture, dual culture and polyculture (the three species in the same pot) for 30 days.

**Results and Discussion:**

Our results show that inoculation with extreme endophytes improved survival by ca. 15 - 35% and biomass by ca. 30 - 35% in all crop species. The most evident increase was when grown in polyculture, except for survival in spinach, where inoculated plants had higher survival only in dual culture. Nutritional quality and the amount of the antioxidant compounds antioxidants increased in all crop species when inoculated with the endophytes. Overall, fungal endophytes isolated from extreme environments such as the Atacama Desert, the driest desert in the world, could be a key biotechnological tool for future space agriculture, helping plants cope with environmental stress. Additionally, inoculated plants should be grown in polyculture to increase crop turnover and space-use efficiency. Lastly, these results provide useful insights to face the future challenges of space-farming.

## Introduction

Modern agriculture faces unparalleled difficulties that will likely worsen in the near future, threatening food security. Global climate change, desertification, salinization, and reduced water availability exert serious restriction for survival and yield in several crops (Lipper et al., 2014; Amorim et al., 2018). Additionally, human population has increased exponentially and is projected to increase up to a 32% by 2050 (Food and Agriculture Organization of the United State of America, 2022). This could result in a scenario where the ever-higher demand for food will not be met using current production methods and cultivated land areas. Thus, the search for new biotechnological tools, such as the use of beneficial microorganisms, to increase tolerance to abiotic stress (Molina-Montenegro et al., 2016; Molina-Montenegro et al., 2020; Acuña-Rodríguez et al., 2022; Baron and Rigobelo, 2022) and the exploration of new areas for agriculture, such as deserts and even space, arise as a response to the global food crisis (Mortimer and Gilliham, 2022; Smith, 2023; Ye et al., 2022).

Besides the use of microorganisms, other agricultural practices, such as growing different crops species together in a polyculture, have been shown to increase nutritional value, diversity, total biomass, stability, and improve space-use efficiency compared to the traditional practice of monoculture (Igbozurike, 1978; Iverson et al., 2014; Florence and McGuire, 2020). Polyculture increases structural diversity above and belowground, allowing a higher photosynthetic efficiency per area, fewer specialist pests, and, as mentioned above, more efficient use of space and soil (Power, 2010; Florence and McGuire, 2020). This is because different root architectures, for example, are less likely to compete for resources since they explore different parts of the soil (see Postma and Lynch, 2012). Although this practice has shown potential for sustainable agriculture, it has seldom been evaluated in terms of net results combined with biotechnological tools (i.e., mutualistic fungi).

New technological advances have made possible ever longer stays in space, long spatial travels, and even the possibility of exoplanetary colonies. Within the solar system, conditions on other planets usually include stressful conditions like extreme temperatures, high radiation, and low oxygen levels (McKay, 2014). If we are to establish self-sustained colonies or long-termed space travels, we require viable crops to provide both food and oxygen for human survival. In this context, crops that can provide a high nutritional turnover will be likely candidates since space restrictions will likely be in place.

As mentioned above, inoculation with beneficial microorganisms, such as root fungal endophytes, has gained much attention as a possible biotechnological tool to increase crop tolerance to stress (Lata et al., 2018; Acuña-Rodríguez et al., 2020). Particularly, root fungal endophytes isolated from plants inhabiting extreme environments can establish successful associations with other unrelated plant species (including crops). Subsequently, endophytes-inoculated crop species showed higher performance and survival than uninoculated plants under drought, salinity, or cold (Azad and Kaminskyj, 2016; Molina-Montenegro et al., 2020; Chen et al., 2021; Acuña-Rodríguez et al., 2022). These beneficial effects could result from an increase in the expression of key genes as well as hormonal and metabolic pathways, including an increase in stress tolerance-related osmolytes (e.g., proline and starch), ecophysiological performance, and protection of membranes, among others (Azad and Kaminskyj, 2016; Dastogeer, 2018; Acuña-Rodríguez et al., 2019; Molina-Montenegro et al., 2020). In fact, several studies have demonstrated that inoculation with endophytes from extreme environments such as Antarctica or Deserts can improve the antioxidant system in native plants as well as in crops to cope with the abiotic stress prevailing in this harsh ecosystems (Ramos et al., 2018; Hereme et al., 2020; Li et al., 2022).

Here, we evaluated the effect of the inoculation with a mix of two fungal endophytes isolated from the roots of the Desert grass *Distichlis spicata* growing in the Atacama Desert, the driest desert in the world and considered analogous to Mars (Sun et al., 2018; Azua-Bustos et al., 2022). Specifically, we assessed the survival, fresh biomass and nutritional quality of three crop species (lettuce, chard and spinach) subjected to exoplanetary conditions growing in monoculture, dual culture and polyculture (individuals of the three crop species simultaneously) in plants with and without the inoculation of the extreme endophytes mix. Finally, we measured antioxidants levels (flavonoids and phenolic compounds) in order to inquire about a potential mechanism by which the fungal endophytes could exert a protective effect on crops when exposed to the astrobiological simulated conditions.

## Materials and methods

### Sampling and endophyte inoculum

Fifty separete tufts of the grass *Distichlis spicata* (Poaceae) were sampled in the locality of “Aguas Blancas” within the hyper-arid core of the Atacama Desert, Chile. Their root system was collected, stored in sealed plastic bags, and put in a cooler. Samples were transported to the laboratory at Universidad de Talca, Talca, Chile, and stored at 4°C until isolation. The study species was selected based on its tolerance to different and simultaneous environmental stressors (Pessarakli and Marcum, 2013) and because it is considered a model to assess different strategies and mechanisms to survive and improve the edaphoclimatic conditions, even under exoplanetary conditions (Rivera Cárdenas et al., 2022).

The roots of *D. spicata* were then cut into pieces and stored in plastic bags for no more than 12 h at 10°C before the isolation of endophytic fungi. The roots were superficially sterilized by successive immersion in ethanol 70% (1 min) and 2% sodium hypochlorite (3 min), followed by washing with sterile distilled water (2 min) (Collado et al., 1996). Then, the roots fragments were plated on Petri dishes containing potato dextrose agar (PDA, Difco, USA) plus chloramphenicol at 100 g mL/1. The Petri dishes were incubated up to 30 days at 18°C. Formed individual colonies were transferred to PDA medium and stored at 4°C until their utilization in the assays.

In order to know the main species of endophytes inhabiting in *D. spicata* roots we conducted a molecular identification. To do this, we amplified the ITS region of DNA, using the ITS3/ITS4 primer pair (Op De Beeck et al., 2014), extracted from actively growing mycelia using the E.Z.N.A. fungal DNA extraction MiniKit (Omega-Biotek). After sequencing, the fragments of forward and reverse sequences were edited using Geneious v5.4 software (Drummond et al., 2011). The sequence of each endophyte isolated was analyzed with MegaBLAST (Basic Local Alignment Search Tool) (http://blast.ncbi.nlm.nih.gov/Blast.cgi) to determine the percentage of maximal identity and total scores with the sequences of that global database. Overall, we found four species of endophytes with more than 5% of frequency of occurrence in root samples (n = 50): *Penicillium fuscuglaucum* (63%), *Penicillium glabrum* (26%), *Alternaria* sp. (6%), and *Eupenicillium* sp. (5%).

Finally, the inoculum used for all experiments consisted of a concentrated mix of spores (5000 spores g^−1^) obtained from the two most conspicuous fungal strains mentioned above (*Penicillium fuscuglaucum* and *P. glabrum*) in a proportion of 1:1. The liquid inoculum-mix (both fungal endophytes plus non-supplemented tap-water as vector for inoculation) was added three times during two weeks (10 ml per individual) to ensure fungal association with the crops. One week after the last inoculation, occurrence of effective symbiosis was corroborated in root samples by staining and light microscope observation in ten randomly selected individuals from each species. Specifically, endophyte infection was checked by counting aniline blue-stained fungal hyphaes in root cross-sections in 10% of the produced crop plants as the percentage of infested roots (Bacon and White, 2000). Complementarily, sterilized root fragments from the selected individuals were plated on Petri dishes containing potato dextrose agar (PDA, Difco, USA) and were incubated for a 15-days at 22°C. Only those set of plants that showed > 95% of infested-roots and positive growing of fungi into the PDA media were considered as “fungal inoculated-plants (E+)”, becoming a set of plants suitable for their use in the subsequent experiment. Lastly, the persistence of the endophyte infection up-to the end of the experiment, was corroborated by counting aniline blue-stained fungal hyphaes in root cross-sections in 5% of the surviving crop plants.

### Experimental setup

Seeds of lettuce (*Lactuca sativa*), chard (*Beta vulgaris* var. *cicla*) and spinach (*Spinacia oleracea*) were germinated from commercial seeds, and ca. 1000 seedlings per each species were obtained. Seedling were put in 50 cc pots containing 50% native soil, 40% sand and 10% peat. This substrate was previously autoclaved to avoid presence of different microorganisms. Seedlings of each species were randomly assigned to the following treatments: 1) monoculture with (E+) or without endophytes (E-), 2) dual culture (target species + one of the other species) with endophytes (E+) or without endophytes (E-), considering all possible combination of species, and 3) polyculture (all three species grown together) with (E+) or without endophytes (E-). We used 5 growth chambers, each containing 30 E+ and 30 E- plants for monoculture, 15 E+ and 15 E- per species in dual culture, and 10 E+ and 10 E- per species in the polyculture treatment. Plants of all treatments (mono, dual and polyculture as well as plants with or without endophytes) were grown inside these different growth chambers set-up with “exoplanetary conditions”, namely: temperature of 2°C, UV-B radiation of 5μW cm^-2^, low water availability (5 ml of tap water weekly per pot), and an hypoxic atmosphere with an average CO_2_ concentration of 4600 ppm (11.5-times higher than earth’s atmosphere).

Plants were kept under this “exoplanetary conditions” for 30 days and at the end of the experiment, we measured final survival and total dry biomass in all plants. In addition, a sub-sampled of three leaves per five plants per each chamber (n = 25 ind., per each crop species, both for M+ and other 25 ind., for M-) were taken at the end of the experiment for nutrient and vitamin content analysis. We measured total protein content, carbohydrates, fiber, Na, K, Ca, Fe, Mg, P, and vitamin C as described by Oliveira et al. (2010) and Arsal-Köse et al. (2019). These analyses were conducted separately for each crop species and under monoculture condition in the “Laboratorio de Suelos”, at the Universidad de Talca, Chile.

### Determination of total flavonoids and phenolics compounds

Total flavonoid was determined spectrophotometrically according to aluminium chloride colorimetric assay method. Briefly, 0.4 mL 5% sodium nitrate was added to 1 mL methanol extract of strawberry fruit in a test tube. For blank reaction, 1 mL methanol was taken instead of methanol extract of strawberry. After 5 minutes, 0.6 mL of 10% AlCl3.6H2O was added to the mixture. At 6th minute, 2 mL of 1M NaOH was added, followed by shaking thoroughly and measuring the absorbance of the solution at 510 nm against the blank sample (Zhishen et al., 1999). The measurements were compared to a standard curve of quercetin solutions and total flavonoids content was expressed as (µg/g FW) quercetin equivalent. Total phenolic compounds were also determined spectrophotometrically following Folin-Ciocalteau method. In short, 0.5 mL of 10% (0.2 N) Folin–Ciocalteau reagent was added to each test tube containing 1 mL methanol extract of fruit sample and 1 mL methanol alone as blank. The test tubes were shaken for 10 s, covered and incubated for 15 min at room temperature. Aqueous 700 mM sodium carbonate (Na2CO3) solution (2.5 mL) was added to each reaction mixture and then vortexed, covered and incubated at room temperature for 2 h. The absorbance of the solution was measured at 765 nm against the blank sample (Ainsworth and Gillespie, 2007). The measurements were compared to a standard curve of gallic acid solutions and total phenolics were expressed as (µg/g FW) gallic acid equivalent.

### Statistical analyses

To assess differences in survival and biomass between uninoculated (E-) and endophytes-inoculated (E+) plants of chard, lettuce and spinach across different culture types (i.e., mono, dual and polyculture), we analyzed data separately for each crop species with generalized mixed effect models (GLMM) using the function *glmer* implemented in the R package *lme4* using a Gaussian distribution with a ‘log’ link-function. The models included the effect of endophyte treatments, culture types and their interaction as fixed factors, while growth chambers used to perform experiments were considered a random factor. To evaluate the significance of the fixed effects, we used Wald Chi-Squared Tests. Then, we implemented estimated marginal means (a.k.a., least-squares means) to perform multiple comparisons of means using *emmeans* and *contrast* functions from the *emmeans* package.

To characterize differences in nutritional quality between E- and E+ plants, we calculated Cohen’s *f* statistics as a measure of effect size using the *cohens_f* function from the *effectsize* package separately for each crop species under monoculture. Positive and negative effect size values indicate an increase and decrease in the nutritional quality caused by endophytes’ presence. Welch’s t-test accompanied estimated effect sizes to infer the significant effects of the endophyte treatments. Likewise, we assessed for differences in antioxidant compounds (i.e., total flavonoids and phenolics) of the three crop species using Welch’s t-tests. All analyses were performed using R v.4.2.2 (R-CoreTeam, 2022).

## Results

**Figure 1:**
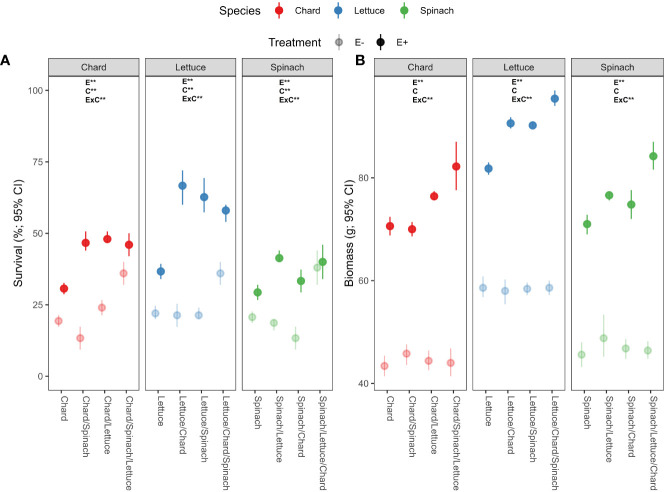
Effects of endophyte treatment (E- and E+), crop culture type (mono-culture, dual- and tri-culture) and their interaction on **(A)** survival and **(B)** biomass percentages in the three crop species, i.e., chard, lettuce and spinach. Mean crop culture types and their 95% confidence intervals (CI) are shown for chard (red), lettuce (blue) and spinach (green) treated without (pale circles) and with endophytes (filled circles). Means are derived from 150 replicate plants per treatment. Asterisks denote significant effects of endophyte treatment (E), crop culture type (C) or their interaction (E×C) as shown in **Table 1**.

There was a significant effects of endophyte treatment (E), crop culture type (C) and their interaction (E×C) on survival percentages in the three crop species (**Table 1A**). In chard and lettuce, endophyte-inoculated (E+) plants showed significantly higher survival percentages than uninoculated (E-) plants across all crop culture types, with the highest differences in dual cultures (**Figure 1A**). In spinach, E+ plants showed significantly higher survival percentages than E- plants just in dual cultures (**Figure 1A**). Regarding biomass, endophyte treatment and its interaction with culture type (E×C) showed significant effects (**Table 1B**). E+ plants showed significantly higher biomass than E- plants across all culture types in the three crop species (**Table 1B**). The highest differences were evidenced for all crop species in polyculture, followed by dual and monocultures (**Figure 1B**).

**Table 1:** Results of GLMMs on a) survival and b) biomass for chard, lettuce and spinach.

a) Survival										
Chard				Lettuce			Spinach			
	χ^2^	DF	*P*	χ^2^	DF	*P*	χ^2^	DF		*P*
Endophytes	20.015	1	< 0.001	19.807	1	< 0.001	8.608	1		< 0.001
Culture	85.858	3	< 0.001	37.698	3	< 0.001	80.802	3		< 0.001
E×C	57.282	3	< 0.001	41.691	3	< 0.001	42.807	3		< 0.001
b) Biomass										
Chard				Lettuce			Spinach			
	χ^2^	DF	*P*	χ^2^	DF	*P*	χ^2^	DF	*P*	
Endophytes	192.447	1	< 0.001	402.252	1	< 0.001	165.515	1	< 0.001	
Culture	1.876	3	0.599	0.381	3	0.944	3.239	3	0.356	
E×C	19.229	3	< 0.001	47.641	3	< 0.001	14.387	3	< 0.01	

Endophytes treatments (E), crop culture type (C) and their interaction (E×C). Chi-square statistic values (χ^2^), degree of freedom and P-values are shown. Significant differences are denoted in red.

**Figure 2:**
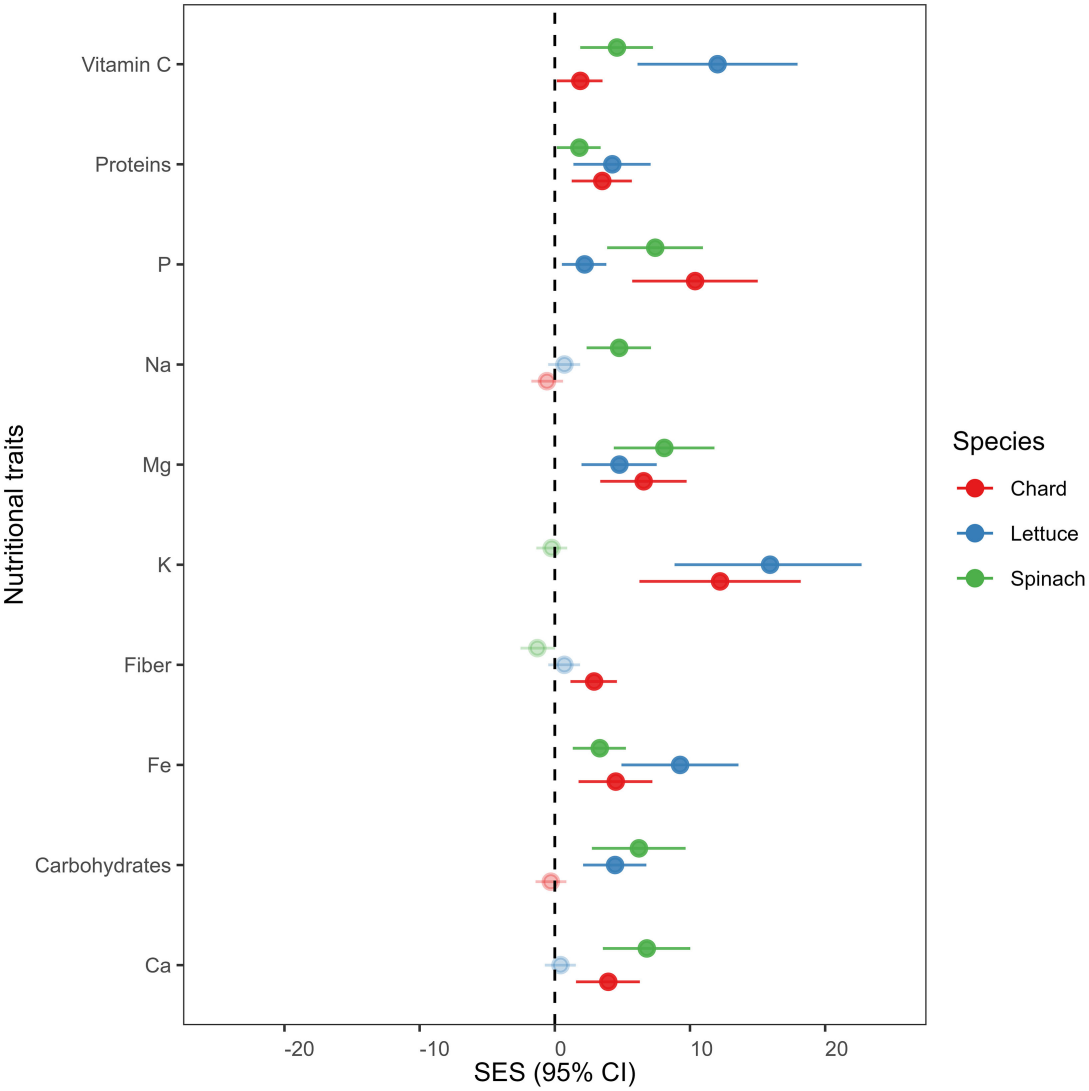
Mean effect sizes (Cohen’s f statistics) of differences in nutritional traits between endophytes-inoculated plants compared to un-inoculated plants in all crop species. Error bars depict 95% confidence intervals (CIs). A mean effect size is significantly different from zero when CIs do not overlap zero. Significant results are denoted with filled circles. Positive (or negative) effect sizes indicate that endophytes-inoculated plants have on average greater (or lesser) nutritional trait concentration than un-inoculated plants. Means are derived from 50 replicate plants per each crop species (25 ind., E+ and 25 ind., E-).

We found that overall nutrient-related traits increased in endophytes-inoculated plants compared to uninoculated plants in all crop species (see **Figure 2**). Vitamin C, proteins, phosphorus (P), magnesium (Mg), potassium (K), fibers, iron (Fe) and calcium (Ca) were significantly higher in E+ than in E- plants of chard. In lettuce, vitamin C, proteins, P, Mg, K, Fe and Carbohydrates increased significantly in E+ plants compared to E- plants. Finally, vitamin C, Proteins, P, Na, Mg, Fe, Carbohydrates and Ca were also higher in E+ compared to E- plants of spinach.

**Figure 3:**
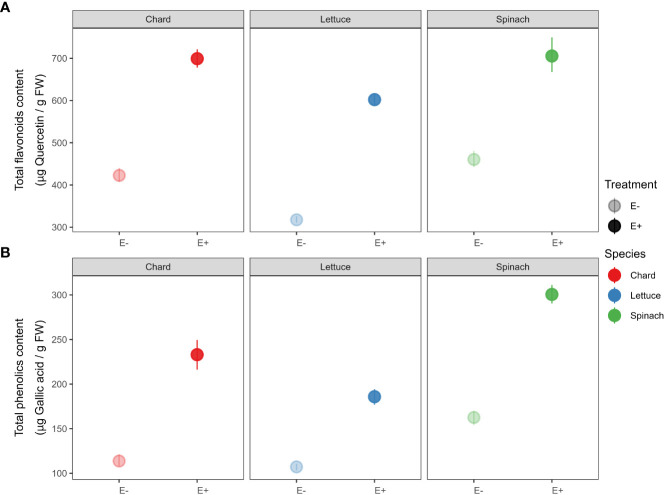
Effects of endophyte treatment –uninoculated (E-) *vs*. endophytes-inoculated (E+)– on **(A)** total flavonoids and **(B)** total phenolics in the three crop species, *i.e.*, chard, lettuce and spinach. Mean and 95% confidence intervals (CI) are shown for E- (pale circles) and E+ (filled circles) plants of chard (red), lettuce (blue) and spinach (green). Means are derived from 50 replicate plants per each crop species (25 ind., E+ and 25 ind., E-).

Similarly, we detected that total antioxidant compounds increased in endophytes-inoculated plants compared to uninoculated plants in all crop species (see **Figure 3**). Specifically, total flavonoids content in E+ plants was significantly higher than plants E- of chard (*t*-statistic = -18.456, *P* < 0.001), lettuce (*t* = -31.672, *P* < 0.001) and spinach (*t* = -10.039, *P* < 0.001). We also found that E+ plants had higher total phenolic content than plants E- of chard (*t* = -12.292, *P* < 0.001), lettuce (*t* = -15.656, *P* < 0.001) and spinach (*t* = -18.984, *P* < 0.001).

## Discussion

Inoculation with mixed root fungal endophytes isolated from a plant species inhabiting the Atacama Desert increased survival, growth (biomass), and nutritional quality in the three tested crops (lettuce, chard and spinach) grown under exoplanetary conditions, notably when grown in polyculture. In addition, the presence of these fungal endophytes increased the production of total antioxidant compounds in all crop species, exerting a biochemical protection against physiological stress. Results support that fungal endophytes are a key biotechnological tool for current and future sustainable agriculture, and they could be essential in establishing exoplanetary colonies in stressful abiotic conditions.

Several studies have confirmed the role of endophytes in stress tolerance, especially to drought and osmotic stress (Azad and Kaminskyj, 2016; Molina-Montenegro et al., 2020; Morsy et al., 2020; Chen et al., 2021), UV-radiation (Ramos et al., 2018), and cold (Acuña-Rodríguez et al., 2022), separately. However, to our knowledge, this is the first study to test the effect of the inoculation with fungal endophytes on tolerance to exoplanetary conditions in crops, which includes a combination of drought, low temperature, low oxygen, and high UV-B radiation. These benefits to the plant are likely due to increased antioxidant capacity, ion homeostasis, hormonal modulation, and activation of key genes involved in stress tolerance (i.e., Tyagi et al., 2022). In fact, drought can result in increased reactive oxygen species (ROS), creating oxidative stress within plant tissues (Farooq et al., 2009; Ahmed et al., 2013). Similarly, high UV radiation can also result in increased ROS (see Nishiyama et al., 2011). Thus, as stated earlier, endophytes that can reduce oxidative stress will likely increase tolerance to drought and radiation stress, as expected in exoplanetary conditions. In this sense, our results are in agreement with this previous finding, since crop species in symbiosis with fungal endophytes deployed higher levels of antioxidant compounds and, in turn, higher performance and nutritional quality. Thereby, fungal endophytes from Atacama Desert seem to be responsible for the better performance of crops under very stressful conditions, avoiding the cellular impairing typically induced when a plants is exposed to abiotic stress (You and Chan, 2015).

As mentioned above, we found increased nutritional quality in the three tested crops when plants were inoculated with endophytes, and noticeable enhanced nutritional quality was obtained in polyculture. Previous studies have shown that other endophytes such as those of *Epichloë* genus can increase the nutritional quality of host plants (Singh et al., 2018; Lin et al., 2020). Polyculture, on the other hand, has been known to increase yield and nutritional quality in crops compared to monoculture (Bainard et al., 2020; Burton and Kemanian, 2022). The combination of both polyculture and endophyte inoculation may act additively or synergistically to increase nutritional quality, but further studies should be conducted to determine this. Nevertheless, the increase in key nutrients and minerals obtained here is highly relevant to increase the nutritional turnover of crops, most relevant in long space travels and potential extraplanetary colonies where the available space will likely be limiting.

In this study, we demonstrated that endophytes isolated from a grass growing in the Atacama Desert, as mentioned earlier one of the harsh places worldwide and considered an analogous of Mars, can form a beneficial interaction with three common crop species. In this context, extreme environments such as Antarctica, high elevation mountains, or deserts could be proposed as an untapped source for extremophiles inhabiting these ecosystems can be used in other target plant species to confer them increased stress tolerance (Rodriguez et al., 2008; Molina-Montenegro et al., 2020), even under exoplanetary conditions. This is mainly due to the fact that, in extreme environments, natural selection should favor those fungal endophytes that confer highest stress tolerance to host plants. Thus, the Atacama Desert can be a key source of microorganisms for bioprospecting, aiming at developing new research avenues for the future “space farming”.

As we continue to improve technology, longer space travels and stays in space stations will likely be more frequent. Even inhabited space colonies in nearby planets could occur in the not-so-far future. Developing methods to produce fresh food in such scenarios is essential, since fresh food is more nutritious and has psychosocial benefits (i.e., increased variety of flavor and texture) over consuming only nutrient capsules and supplements (Zwart et al., 2009; Polivkova et al., 2010; Perchonok et al., 2012). Here we show that beneficial microorganisms, extreme fungal endophytes in particular, could be a possible biological tool to meet, along with the advances in irrigation and illumination, the challenges of the agriculture in exoplanetary conditions (see Monje et al., 2002). Additionally, research on space farming and crop production under exoplanetary conditions could also produce solutions to current agricultural problems on Earth related to the devastating effects of global climate change.

## Data availability statement

The raw data supporting the conclusions of this article will be made available by the authors, without undue reservation.

## Author contributions

CA and MM-M conceived the ideas; CA and MM-M compiled data; VME generated figures and performed the statistical analyses; CA, VME, and MM-M wrote the draft manuscript, and all authors contributed to editing it. All authors contributed to the article and approved the submitted version.
